# Targeting RNA N6-methyladenosine modification—— a novel therapeutic target for HER2- positive gastric cancer

**DOI:** 10.3389/fonc.2024.1387444

**Published:** 2024-06-20

**Authors:** Lijun Jia, Di Zhang, Xiaoman Zeng, Li Wu, Xiaowei Tian, Na Xing

**Affiliations:** Department of Anesthesiology, Pain and Perioperative Medicine, The First Affiliated Hospital of Zhengzhou University, Zhengzhou, China

**Keywords:** HER2-positive gastric cancer, n 6 -methyladenosine modification (m6A), glycolysis regulation, therapeutic efficacy, survival rate

## Abstract

Gastric cancer is one of the most common cancers and is considered the 5^th^ most frequent occurring cancer worldwide. It has gained great attention from the clinicians and researchers because of high mortality rate. It is generally treated with chemotherapy, radiotherapy, and surgery. Recently, additional treatment options including immunotherapy and targeted therapy and immunotherapy have been developed. However, poor prognosis, limited survival rate of patients, and drug resistance to treatment remain critical problems. To improve treatment options or to overcome the bottleneck of treatment, identification of diagnostic and prognostic markers, determining the most effective therapeutic options, and uncovering the molecular regulations associated with treatment strategies are required. In this regard n^6^-methyladenosine (m6A) regulation is considered important. This reversible modification plays a crucial role in progression, development and treatment of *HER2*-positive gastric cancer. Here, we discuss the role of m6A modification in *HER2*-positive gastric cancer progression through collecting related studies at present. We further discuss the association of m6A modification with therapeutic efficacy in *HER2*-positive gastric cancer and list some examples. We conclude that modification of m6A can be a new strategy for improving the prognosis and survival rate of *HER2*-positive gastric cancer patients.

## Introduction

The incidence of gastric cancer ranks fifth among all malignant tumours and is one of the most serious cancer types that poses huge health burden worldwide. A high mortality rate has been recorded in patients with gastric cancer, making it the third death causing cancer (1). Several invasive and no invasive treatment options have been developed, however promising treatment option that could equally cure all patients with gastric cancer is yet to develop. In order to relate molecular features of gastric cancers to clinical features and histological phenotypes, several molecular classifications have been attempted. The results from an integrated genome-wide analyses “The Cancer Genome Atlas (TCGA)” related to alterations of DNA copy number, mRNA mutations, and patterns of proteins, were published, where four distinct subtypes of gastric cancer at molecular level were proposed (2). The molecular level investigations have increased our knowledge, however testing for additional molecular subgroup still needs to be done. In this regard *human epidermal growth factor receptor-2* (*HER2*) has been known an important factor in gastric cancer, which is being tested regularly because of it is a strong predictive biomarker (3). Its expression in gastric cancer is regard *HER2*-positive gastric cancer, and is considered the most serious cancer type, with limited therapeutic efficacy because of therapeutic resistance. It is different than other gastric cancer types because of the expression pattern or levels of *HER2* gene. As *HER2* gene is altered in these gastric cancers, therefore, developing therapeutic options targeting molecular or immunological pathways can improve therapeutic efficacy (4–6). The varying regulatory processes in HER2-positive gastric cancer, makes the diagnosis and treatment more complicated. The treatment options for *HER2*-positive gastric cancer have been developed from chemoradiotherapy and immunotherapy to targeted therapy, but these options are limited by the risks of recurrence, metastasis, and resistance. Moreover, the lack of obvious diagnostic and prognostic markers makes the evaluation of efficacy difficult. Therefore, further investigations on diagnosis and therapeutic options are necessary in order to improve the prognosis and survival of *HER2*-positive gastric cancer patients. m6A a methylation modification, is a well described epigenetic modifications in mRNAs of eukaryotes (7). The prominent associated genes with m6A (writers, erasers and readers) contribute to the posttranscriptional regulation of stability, translation, and splicing of RNA (8). Among the common m6A methylation mediated health conditions, cancer is considered the most relevant disease, where tumor proliferation, invasion, progression, metastasis and therapeutic processes are regulated by M6A methylation (9, 10). *HER2*-positive cancer is a complex process of epigenetic alterations (11), and it is deemed necessary to understand role of m6A methylation in therapeutic efficacy. This paper describes the key regulatory relationships between m6A modification, and treatment strategy and glycolysis in *HER2*- positive gastric cancer.

## Regulators of m6a modification

**Figure 1:**
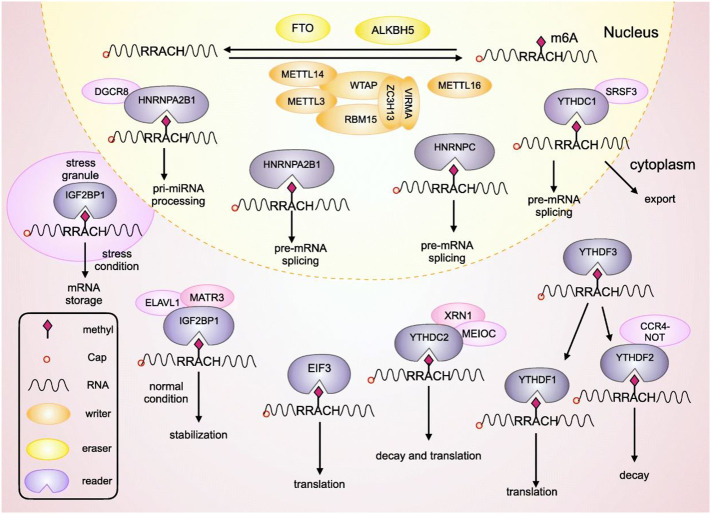
This figure shows LHPP mediated the regulation of gastric cancer. The growth of gastric cancer was induced in response to LHPP knockdown and inhibited by overexpression of LHPP. **(A)** Tumour size was measured at different time points as depicted in this figure. Hematoxylin-and-eosin staining shows the overexpressed LHPP reduced. **(B)** gastric cells were injected intraperitoneally followed by recording and examination of peritoneal metastasis. **(C)** Methyltransferase like 14 mediated m6A modification of LHPP mRNA represses the expression of LHPP, which in turn inhibits the phosphorylation of GSK3b that leads to the mitigation or inhibition of metastasis, invasion, glycolysis, and proliferation of gastric cancer cells. This figure was adopted and modified from the paper published by Lin et al, (15) in Cell Death & Disease according to the rights and permissions (https://creativecommons.org/licenses/by/4.0/) from the copyright holder.

M6A is a well-known methylation modification on the sixth N atom of adenine. It is one of the most common methylation modifications in noncoding RNA as well as mRNA in mammalian systems, and are commonly present in 3’ untranslated regions of RNA or near stop codons (12). These reversible modifications are involved in RNA degradation, splicing, and translation, with the help of writers, erasers and readers (13). This m6A methylation is the most prevalent modification on eukaryotic mRNA. Earlier reports indicate that m6A modulates gene expression to further regulate cellular processes including differentiation, self-renewal, apoptosis and invasion. Generally, m6A is recognized by reader proteins, installed by methyltransferases, and removed by m6A demethylases (14). The detailed regulatory mechanism can be seen in **Figure 1**. The m6A writers include methyltransferases-like 3, methyltransferase-like 14, RNA binding motif protein, and Wilms’ tumour 1-associating protein. The three methyltransferases-like 3, methyltransferase-like 14, and Wilms’ tumour 1-associating protein constitute a complex known as methyltransferase complex, which contribute the methylation process (16). Methyltransferase-like 3 has been detected as an oncogene that promotes progression of gastric cancer and colorectal cancer, however, it inhibits the progression and development of hepatocellular carcinoma. Demethylation activating molecules m6A erasers (fat mass and obesity-associated and alkB homologue 5), are regulated by writers. Both fat mass and obesity-associated and alkB homologue 5 can act either as tumour suppressors or oncogenes in different cancer types (13, 16). Furthermore, in the process of M6A modification m6A readers (heterogeneous nuclear ribonucleoproteins, insulin-like growth factor 2 mRNA-binding proteins and YTH domain family proteins) can directly recognise and bind to the modification site. These readers contribute to the splicing as well as nuclear export of mRNA to promote the translation and degradation of target mRNA. High expression of YTH domain family proteins has been found to be associated with poor prognosis of gastric cancer (13, 16, 17).

## RNA methylation in HER2-positive gastric cancer

HER2-positive advanced gastric cancer managed with targeted therapies, chemotherapy, immunotherapies, as well as combinational therapies. The m6A eraser oncogenes (such as Fat mass and obesity-associated protein termed as FTO) have found to be upregulated in gastric cancer tissues, and this expression of FTO has been detected to play crucial role in differentiation and lymph node metastases. FTO inhibits SRSF2 binding, thereby controlling mRNA splicing and regulates adipogenesis. It also blocks the mRNA degradation mediated by YTHDF2 by reducing m6A levels of cyclin-dependent kinase 2. This activity of FTO finally leads to promoting progression and adipogenesis of fat cell cycle. Moreover, FTO can regulate leukemogenesis by modulating differentiation, apoptosis and proliferation of acute myeloid leukemia cells (7, 14). Increased expression of FTO is significantly linked with poor prognosis of gastric cancer, while its downregulation inhibits the migration, invasion and proliferation of gastric cancer cell lines *in vitro* (18). Reduced modification of m6A can be implicated into the prediction of malignant phenotypes and Wnt-Akt signaling pathways in *HER2*-positive gastric cancer (16, 19). Zhang et al, generated a proteomics-based GC cohort and the TCGA-GC cohort, and merged the expression of m6A writers (W), readers (R), and erasers (E), to represent the modification of m6A by W, R, and E signatures in gastric cancer. The patients were stratified according to these signatures in order to decipher the associations between m6A and prognosis, clinical indices as well as critical mutations. They predicted and validated the biological function of m6A in GC by gene set enrichment analysis and *in vitro* experiments, respectively. Zhang et al, detected W and R as potential tumor suppressors and E as a potential oncogene. Patients with decreased m6A modification depicted higher mutations of cancer associated genes such as *Cadherin 1* (*CDH1*)*, GLI3, Ras homolog gene family, member A* (*RHOA*)*, Mucin 6, Oligomeric Mucus/Gel-Forming* (*MUC6*), and *TP53*), well as exhibited worse clinical outcomes. The suppression of m6A activated Wnt-PI3KAkt signaling pathway to promote proliferation and invasiveness, while these changes were successfully reversed by the upregulation of m6A. These observations indicated that m6A modifications can play crucial role in immunological regulations, immune response and interferon signaling in gastric cancer (19, 20). Therefore, targeting the erasers or writers can help in developing promising therapeutic strategies or discover novel treatment options for the treatment of *HER2*-positive gastric cancer.

## Non-coding RNA regulation by m6A methylation

The modification of m6A modulates the regulatory processes of non-coding (nc)RNA such as splicing and maturation. The modified m6A regulates readers binding to lncRNAs, which play a crucial role in the processes of RNA transcription and post-transcriptional modification (21). Moreover, the interaction between lncRNA and specific DNA sites are also regulated by m6A modification (22). Methylation modification of methyltransferases-like 3/14 is positively correlated with lncRNA metastasis-associated lung adenocarcinoma transcript 1 (MALAT1), which is regulated by m6A in alternative splicing as well as in promoting post-lncRNA’s expression (23). Methyltransferase of U6 spliceosome small nuclear RNA (snRNA) methyltransferases-like 16 interacts with MALAT1 triple helix and promotes its cleavage. ALKBH5 promotes tumorigenesis by reversing the methylation of nuclear para-peckle assembly transcript 1 (NEAT1), indicating a critical role of m6A modification in gastric cancer, while lncRNA differentiation antagonizing nonprotein coding RNA (DANCR) plays crucial role in cancer progression and development (24, 25).

M6A modification regulate maturation of miRNAs, which contribute to translational inhibition or mRNA degradation (26). Methyltransferases-like 3 regulates the maturation of miR-221/222 and promotes the cleavage of pri-miRNA-let-7e. Similarly, methyltransferases-like 14 is associated with maturation of tumor suppressors miR-375, miR-19a, and miR-126, which in turn can inhibit the migration and invasion gastric cancer (25, 27). In addition to “writers” several readers can recognize m6A-modified sites to promote miRNA maturation with the help of modified regulatory proteins. In progression of cancer, over-expressed m6A modification of methyltransferases-like 3 leads to over-maturation of miR-25, which then contribute to cancer progression (28). Furthermore, m6A modification can influence the function of circRNAs, which are competitive endogenous RNAs and can sponge miRNA. Earlier reports have indicated that m6A modification can influence the translation process of circRNAs having protein coding potential (25). In the next following parts, we would like to introduce some new findings in gastric cancer and highlight some promising therapeutic target and mechanism for Her2-positive gastric cancer.

## M6A modification may increase therapeutics efficacy against HER2-positive gastric cancer through regulation of glycolysis

The importance of m6A modification can be depicted from the findings that it regulates translation, nuclear export and RNA processing (29). For promoting cancer metastasis and progression and metastasis, metabolic alterations play crucial role, where regulating carbon is important. Studies have shown that enhanced glycolysis in response to upregulation glycolytic genes in gastric cancer can induce glycolytic phenotype and increase glucose dependence. This glycolysis regulation plays crucial role in gastric cancer progression and development (30). To direct carbon flux into glycolysis from oxidative phosphorylation and regulate the development of obesity m6A is a key regulator, which can mediate the expression of UCP2 and PNPLA2 proteins expression to regulate obesity development. Knockdown of methyltransferase 3 suppresses lactate production, glucose consumption, ATP generation, and downregulates genes associated with glycan degradation and glycolysis (30, 31). ATP generation and glycolysis are critically associated with cancer progression, metastasis, development, therapeutic efficacy and resistance (32). Earlier reports indicated that deletion of methyltransferase like 3 can suppress the growth rate and proliferation, however, this effect can be reversed by the overexpression of PDK4. Moreover, overexpressed PDK4 can reduce the sensitivity of doxorubicin, which is generally increased by the deletion of Mettl3 methyltransferase like 3. These observations indicate that methyltransferase like 3 mediated growth and drug sensitivity is also regulated by PDK4. Li et al, reported that PDK4 can decrease the suppression effect of methyltransferase like 4 on tumor growth, however, the depletion of methyltransferase like 3 downregulates PDK4, suggesting that the expression of PDK4 is regulated by methyltransferase like 3 enzyme (30).

Effective coordination between cancer cells and glycolysis as well as glutamate decomposition is required to meet their requirements proliferation and survival. In the case of gastric cancer, the metabolic adaptations and mechanisms of tumour proliferation, metastasis, and invasion need to be fully elucidated for developing promising and efficient therapeutic intervention methods (33, 34). In this regard, a histidine phosphatase “Phospholysine phosphohistidine inorganic pyrophosphate phosphatase (LHPP)” can be considered for its role in tumor progression (35). Lin and colleagues investigated the role of LHPP in the metastasis and invasion of gastric cancer *in vitro* and found that knockdown of LHPP expression can induce proliferation, while upregulating the expression of reduces the proliferation ability (36–40). Further experiments depicted that downregulation or knockdown of LHPP induces the abilities of gastric cancer cells to invade and migrate. On the other hand, overexpression or upregulation of LHPP was found to decrease the invasion and migration potential of gastric cell lines. Moreover, *in vitro* study showed that knockdown of LHPP causes increased drug resistance, while overexpressed LHPP decreases drug resistance of GC cells. These results were further verified *in vivo*, by using a subcutaneous tumour mouse model. The obtained results indicated that overexpression of LHPP leads to inhibit metastasis and tumour growth as detected by decelerated growth in tumour volume. Overall, these results indicated that LHPP has the potential to suppress tumorigenicity of gastric cancer by inhibiting invasion, metastasis, and proliferation of gastric cancer cells and reducing therapeutic resistance (15). It is noteworthy that the tumour suppression effect of LHPP is induced by acetylation, which in turn is regulated by phosphatase inhibitors. LHPP plays an important role in regulation of cell energy metabolism pathways, Wnt ignalling pathway, and Akt signalling pathway, which in turn play crucial role in metabolism of cancer cells such as hyperactivated Akt promotes aerobic glycolysis. LHPP can significantly inhibit aerobic glycolysis in gastric cell by inhibiting glycolysis-related proteins, where LHPP is negatively related with hypoxia-inducible factor (HIF)1A. Knockdown or overexpression of LHPP decreases or increases the cell oxygen consumption rate respectively, suggesting that LHPP has the potential to inhibit aerobic glycolysis. LHPP mediated inhibition of Wnt pathway leads to the inhibition of gastric cancer cells (15, 36). The aforementioned details indicate that m6A plays an important role in gastric cancer metabolism, thus m6A modification can be targeted to develop novel therapeutic strategies against HER2-positive gastric cancer.

## m6A can be regulated or targeted using immunotherapeutics

N6 -Methyladenosine (m6 A), methylated adenosine at the N6 position, is a widespread and abundant modification in messenger RNA (mRNA) and non-coding RNAs (ncRNAs), and represents one of the well-studied RNA modifications thus far.

N6 -methyladenosine influences almost every stage of mRNA metabolism, including RNA folding and structure (37), maturation (41, 42), nuclear export (43, 44), translation (45, 46), and decay (29), as well as other RNA modifications, such as adenosine-to-inosine editing (47, 48). As the most common internal mRNA modification found in eukaryotes, m6A modification is widely implicated in multiple biological processes, such as circadian rhythm (49), adipogenesis (50), spermatogenesis (51), embryonic stem cell self-renewal and differentiation (52), cortical neurogenesis (52, 53).

Numerous investigations have demonstrated that Wnt/b-catenin signaling contributes to the primary resistance to immunotherapy by influencing tumor-cell functions and immune surveillance (11). Furthermore, b-catenin activation has been linked to Treg infiltration (53), T-cell rejection, and resistance to anti-PD-L1/anti-CTLA-4 immunotherapy (54). It has also been documented that the crosslink between cancer cells and tumor-associated macrophages (TAMs) is mediated through Wnt/b-catenin signaling (55). Thus, we conclude that inhibiting the Wnt/b-catenin pathway may hold immense potential as a possible adjuvant for immunotherapy. The Wnt/b-catenin signaling pathway can be regulated by m6A modifications, and then facilitate chemoresistance in various cancers.

Apart from the aforementioned two pathways, m6A influences the pathogenesis and progression of cancer through additional classical pathways, such as p53, EMT, mitogenactivated protein kinase (MAPK), PI3K/AKT/mammalian target of rapamycin (mTOR), and p38/extracellular signalregulated kinase (ERK). Activation of the PI3K/AKT/mTOR pathway has been linked to the tumor immune microenvironment and PD-L1 expression, according to reports (56), implying a novel indication for cancer immunotherapy.

## Regulation of m6A using nanomaterials

Nanoparticles (NPs) platforms have emerged as promising carriers in cancer therapy (31). An increasing amount of evidence suggests that epigenetics plays a crucial role in the initiation and progression of different types of diseases. To alter biological activity, NMs may cycle between methylating specific genes and the entire genome. A possible mechanism for the harmful effects of NMs could possibly involve abnormal up- or down-regulation of ncRNAs.

In recent years, scientists have developed NPs that can precisely and efficiently deliver mRNAs, siRNAs, and protein-based drugs into tumor cells (50–56). Recent research in HCC found that METTL3 can stabilize the RNA transcript of a long non-coding RNA-LINC00958 via m6A modifcation, and aberrant overexpression of LINC00958 is an important cause of accelerated HCC. Moreover, specifcally delivering NP-encapsulated siRNA of LINC00958 to tumor cells in the TME reduced m6 A modifcation in LINC00958 and inhibited the progression of HCC.

For targeted delivery of drugs into tumors cells, many kinds of NPs, such as lipid-based NPs (47), polymer-based NPs (48), and inorganic NPs (49), can be recognized by TAMs and then deliver drugs or RNA into TAMs of the TME. For example, C-C motif chemokine receptor 2 siRNA-loaded lipid NPs prevent the recruitment of TAMs into the tumors. Polymeric NPs can be loaded with siRNAs to target vascular endothelial growth factor and placental growth factor signaling in both tumor cells and M2 TAMs, skewing the immunosuppressive M2 TAMs to the M1 type and thereby inhibiting tumor growth. Recent studies found that METTL14, METTL3, and their target genes, Spred2 and Irakm, in macrophages are associated with tumor progression (52–56). Therefore, targeted delivery of NP-encapsulated Mettl3, Mettl14, or Spred2 mRNA or of Irakm siRNA into TAMs might promote TAM polarization, reduce Treg cell infiltration, promote the cytotoxic function of CD8+ T cells, and reverse immune suppression in the TME.

## Conclusion and future prospects

HER2-positive gastric cancer is one of the tumours with a large diagnostic and therapeutic complications, and greater adverse reactions. The quality of patient’s life has been largely improved through a number of improvements and developments in surgical procedures, endoscopic resection techniques, immunotherapy, chemotherapy, and targeted therapy. However, a number of serious obstacles including high mortality rate, drug resistance especially anti HER2 resistance, and diagnostic complications are yet to be addressed for further improvements in the life of HER2-positive gastric cancer patients. Recently, m6A modification has been studied widely and is being modified in different models to be considered for the treatment gastric cancer. Exploring the mechanisms of drug resistance related to m6A modification in HER2-positive gastric cancer requires higher attention from the researchers. In this review, we discussed the role of m6A modification in prognosis and drug resistance in HER2-positive gastric cancer. Different modulators for m6A modulators (such as methyltransferases) and FGFR4 can be used as targets for different therapeutic options such as immunotherapeutic and chemotherapeutic. Moreover, m6A-modified RNA can also be targeted for developing advanced therapeutic options against HER2-positive gastric cancer. Researchers are currently focusing on exploring how m6A modification patterns can be used as prognostic, diagnostic, and predictive biomarkers for cancer. Identification of specific m6A modifications, personalized treatments and non-invasive diagnostic options can be developed. It is evident that dysregulated m6A modification contributes to gastric cancer, indicating that m6A regulators (enzymes) can be targeted to develop novel therapeutic strategies. In this regard, m6A levels modulating small molecules can be designed for tumor progression inhibition, and therapeutic options can increase the efficacy of currently available treatment options. Researchers can use the phenomenon “modulating the modification of m6A levels by targeting m6A regulators” to develop approaches based on precision medicine according to the molecular profile of individual patients.

Genes related to regulation of m6A can be related to onset, development and progression of HER2-positive gastric cancer thus can be used as prognostic markers. Novel drugs developed on the basis of targeting m6A modification to inhibit methyltransferase like 3 and other molecules have shown tremendous anticancer effects. Therefore, using m6A to develop advanced therapeutic options can help in overcoming drug resistance, thereby improving treatment outcomes in patients with HER2-positive gastric cancer. Overall, translation of m6A modification into clinical research system can help in advancing precision medicine, personalized medicines, and therapeutic strategies for gastric cancer especially HER2- positive gastric cancer. However, prior to translate into clinical research, the molecular mechanism and associated complications at molecular level should further be explored.

## Data availability statement

The raw data supporting the conclusions of this article will be made available by the authors, without undue reservation.

## Author contributions

LJ: Conceptualization, Methodology, Data curation, Investigation, Supervision, Visualization, Writing – original draft, Writing – review & editing. DZ: Conceptualization, Methodology, Visualization, Writing – original draft, Writing – review & editing. XZ: Conceptualization, Methodology, Visualization, Writing – original draft, Writing – review & editing. LW: Conceptualization, Methodology, Data curation, Investigation, Visualization, Writing – original draft, Writing – review & editing. XT: Conceptualization, Methodology, Data curation, Investigation, Visualization, Writing – original draft, Writing – review & editing. NX: Conceptualization, Methodology, Supervision, Visualization, Writing – original draft, Writing – review & editing.
